# Proteasome inhibition mediates p53 reactivation and anti-cancer activity of 6-Gingerol in cervical cancer cells

**DOI:** 10.18632/oncotarget.6383

**Published:** 2015-11-25

**Authors:** Namrata Rastogi, Shivali Duggal, Shailendra Kumar Singh, Konica Porwal, Vikas Kumar Srivastava, Rakesh Maurya, Madan L.B. Bhatt, Durga Prasad Mishra

**Affiliations:** ^1^ Cell Death Research Laboratory, Division of Endocrinology, CSIR-Central Drug Research Institute, Lucknow, India; ^2^ Department of Radiotherapy, King George Medical University, Lucknow, India; ^3^ Department of Host Defense, WPI Immunology Frontier Research Center, Osaka University, Suita, Osaka, Japan; ^4^ Medicinal Process Chemistry Division, CSIR-Central Drug Research Institute, Lucknow, India

**Keywords:** proteasome, p53 reactivation, cervical cancer, HPV, 6-Gingerol, Chromosome Section

## Abstract

Human papilloma virus (HPV) expressing E6 and E7 oncoproteins, is known to inactivate the tumor suppressor p53 through proteasomal degradation in cervical cancers. Therefore, use of small molecules for inhibition of proteasome function and induction of p53 reactivation is a promising strategy for induction of apoptosis in cervical cancer cells. The polyphenolic alkanone, 6-Gingerol (6G), present in the pungent extracts of ginger (Zingiber officinale Roscoe) has shown potent anti-tumorigenic and pro-apoptotic activities against a variety of cancers. In this study we explored the molecular mechanism of action of 6G in human cervical cancer cells *in vitro* and *in vivo*. 6G potently inhibited proliferation of the HPV positive cervical cancer cells. 6G was found to: (i) inhibit the chymotrypsin activity of proteasomes, (ii) induce reactivation of p53, (iii) increase levels of p21, (iv) induce DNA damage and G2/M cell cycle arrest, (v) alter expression levels of p53-associated apoptotic markers like, cleaved caspase-3 and PARP, and (vi) potentiate the cytotoxicity of cisplatin. 6G treatment induced significant reduction of tumor volume, tumor weight, proteasome inhibition and p53 accumulation in HeLa xenograft tumor cells *in vivo.* The 6G treatment was devoid of toxic effects as it did not affect body weights, hematological and osteogenic parameters. Taken together, our data underscores the therapeutic and chemosensitizing effects of 6G in the management and treatment of cervical cancer.

## INTRODUCTION

Cervical cancer is one of the most prevalent gynecological cancers among women in developing countries like India [1]. Epidemiologically, cervical cancer is associated with high-risk human papilloma virus (HPV) infection [2]. HPV 16 and 18 are the most common strains associated with genomic integration and cervical cancer [3, 4]. The HPV encoded oncoproteins (namely E6 and E7), induce immortalization and transformation of primary cervical keratinocytes involved in tumor progression [5].

In spite of the improved diagnostic methods for early detection and vaccination, recent records suggest growing numbers of cases of cervical cancer globally [1, 3]. At present prophylactic vaccination and chemotherapy are the major tools available for therapeutic intervention in cervical cancer [6]. However, major hurdles in the prophylactic usage of vaccination impeding its mass scale implementation are, lack of specificity for few HPV strains, latency of infection and redundancy of other non-vaccinated strains of HPV [2, 6]. Similarly, chemotherapy also suffers from several bottlenecks such as requirement of high doses, severe side effects related to toxicity, activation of pro-survival pathways upon long term exposure and development of chemoresistance [7, 8]. Therefore, it is imperative to look for alternate therapeutic strategies for management of cervical cancer.

Inactivation of tumor suppressor p53 is characteristic of a majority of human malignancies including cervical cancer [9]. In cervical cancer, inactivation of p53 is attributed to the E6 oncoprotein, which binds to the E3 ubiquitin ligase E6-AP and facilitates the proteasomal degradation of p53 [9]. Restoration of p53 function is consequently critical for the effective therapeutic targeting and management of cervical cancer [10]. Circumstantially, a possible approach for p53 reactivation in these cells is either through the suppression of viral proteins expression and function or prevention of the proteosomal degradation of p53 [11].

Proteasomes are large multicatalytic units engaged in non-lysosomal degradation of intracellular proteins [12, 13]. The aberrant proteosomal activation is associated with several cancers including cervical cancer [14] is attributed to the higher metabolic activity, making it a plausible therapeutic target [15]. Several reports suggest that the increased proteasome activity upregulates pro-survival pathways and drug resistance thereby helping in cancer progression [13]. Although synthetic proteasome inhibitors like Bortezomib, have been widely used in cancer therapeutics [16, 17], induction of toxicity and chemoresistance are major concerns limiting its therapeutic value [17]. Therefore, naturally derived proteasome inhibitors like Withaferin A, Celastrol and EGCG are perceived as attractive alternatives and chemotherapeutic agents against a variety of cancers [18, 19].

(6)-Gingerol (1-[4′-hydroxy-3′-methoxyphenyl]-5-hydroxy-3-decanone) (6G), one among the major pungent extracts of ginger (Zingiberofficinale Roscoe, Zingiberaceae), is a polyphenolicalkanone and has established anti-inflammatory and anti-tumorigenic activities [20]. 6G is known to exert its activity through the inhibition of iNOS, suppression of IkBα, nuclear translocation of NFkB, release of cytochrome c, caspase activation, increase in the expression of Apaf-1,induction of oxidative stress, DNA damage, autophagy induction and activation of tumor suppressor proteins including p53 and p21 [21-27] leading to apoptosis. The natural origin, anti-oxidative potential, bioavailability, ease of metabolism and inexpensive nature are favorable attributes of 6G as a promising chemotherapeutic agent against a variety of cancers [20]. However, its precise anticancer effects and the associated mechanism of action in cervical cancer cells are still unknown.

In the present study, we report for the first time that the polyphenolic alkanone 6G, inhibits the proteasome, induces p53 reactivation and apoptotic cell death in cervical cancer cells. 6G also potentiates the cytotoxic effects of the traditional chemotherapeutic drug cisplatin. Taken together our findings suggest that 6G may possibly be used as a single agent or in combination with standard chemotherapeutic drugs as a promising therapeutic strategy for the management and treatment of cervical cancers.

## RESULTS

### 6G inhibits proliferation and induces apoptosis in HPV positive cervical cancer cells

**Figure 1:**
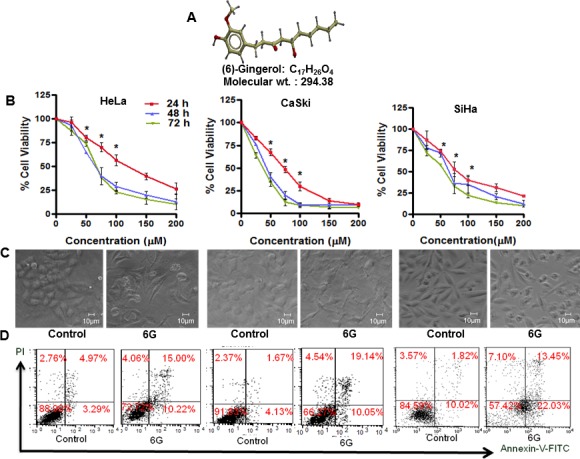
6G inhibits proliferation of HPV positive cervical cancer cells **A.** The 3-Dimensional structure of (6)-gingerol. **B.** HeLa, CaSki and SiHa cells were treated with the indicated concentrations of 6G for 24, 48 and 72h and subjected to cell viability assay using MTT. Dose response curves indicated significant reduction of cell viability in comparison to normal control (*p < 0.05). **C.** Changes in the morphology of cells treated with 6G (50 μM) was detected by phase contrast microscopy. **D.** HeLa, CaSki and SiHa cells were treated with 6G (50 μM) for 24h and subjected to Annexin-V-FITC/propidium iodide staining to determine percent apoptosis. Representative picture presenting percentage of apoptotic cells in each quadrant. Data presented as mean ± SD and are representative of three independent experiments.

HPV infection is associated with majority of the cervical cancers [5]. Therefore, we evaluated the effect of 6G (Figure 1A) on HPV positive cervical cancer cell (HeLa, CaSki and SiHa) viability using the MTT assay. 6G induced a dose and time dependent inhibition of cell viability in all the three cell lines (Figure 1B). At a concentration of 50μM, 6G induced significant inhibition of growth and proliferation of HeLa (20%), CaSki (23%) and SiHa (28%) cells after 24h of treatment. Microscopic analysis of the morphological changes upon 6G treatment further confirmed these results (Figure 1C). Next we assessed the selectivity of 6G by exploring its effects in non-transformed cells like HACAT, HEK-293, and human PBMCs. The results showed that the 50μM dose of 6G did not induce significant cytotoxicity in these cells (Supplementary Figure S1). Therefore, this dose was selected for further mechanistic studies. In order to determine the type of cell death induced by 6G, Annexin-V-FITC and propidium iodide dual staining based flowcytometry was used for detection of the percent apoptotic or necrotic cells. 6G increased the percentage of apoptotic cells in both early and late apoptotic phases in HeLa (25.22%), CaSki (29.19%) and SiHa (35.48%) cells indicating apoptotic cell death (Figure 1D). On basis of these findings we conducted detailed mechanistic studies using HeLa (HPV-18 positive) and CaSki (HPV-16 positive) cells. Collectively, these results suggested that 6G significantly inhibits proliferation through apoptotic cell death in cervical cancer cells.

### 6G induces p53 reactivation independent of HPV oncoprotein inhibition

**Figure 2:**
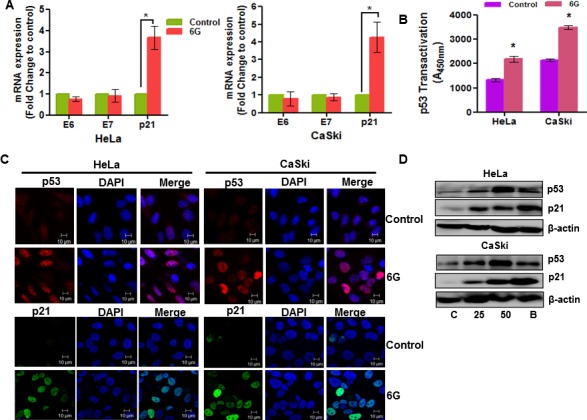
6G induces p53 reactivation in cervical cancer cells **A.** Effect of 6G (50 μM) on the expression of HPV oncogenes (E6 and E7) and p21 was analyzed through real time PCR expression analysis where 18S RNA was used for normalization (*p < 0.05). **B.** Increase in the p53 content was measured by p53 transactivation assay in HeLa and CaSki cells treated with 6G for 18h in comparison to non-treated controls. (*p < 0.05) Data presented as mean ± SD and are representative of three independent experiments. **C.** Expression and localization of p53 (red) and p21 (green) in HeLa and CaSki cells treated with 6G (50 μM) for 18h was assessed by immunofluorescence and confocal microscopy. Images were captured under 63x magnification and are representative images of three independent experiments are presented. **D.** High expression of p53 and p21 proteins in HeLa and CaSki cells treated with 6G (25 and 50 μM) were detected by western blot. Bortezomib (10 nM) treated cells were used for positive control and β-actin was used as loading control. The representative data of three independent experiments is presented.

Transciptional silencing of HPV oncoproteins, E6 and E7 is known to inhibit proliferation of cervical cancer cells [5]. Many natural compounds exert their anti-tumor activity on cervical cancer cells through inhibition of the E6 and E7 proteins [28]. Therefore, we next checked the effect of 6G treatment on the mRNA levels of the E6 and E7 oncoproteins using real time PCR analysis. It was found that 6G treatment did not affect the expression of E6 and E7 mRNA levels in HeLa and CaSki cells (Figure 2A). 6G is known to induce both p53 dependent and independent apoptosis in cancer cells [22, 23]. Moreover, p53 dependent apoptotic pathways are mediated through its downstream target of p21 [26]. So we next assessed the p21 mRNA levels upon 6G treatment. Interestingly, p21 mRNA levels were significantly increased in both the cell types indicating the involvement of p53 dependent apoptosis in these cells (Figure 2A). Furthermore, the increased p53 transactivation upon 6G treatment (for 18h) confirmed the functional restoration of the p53 (Figure 2B). Consistent with these results the immunofluorescence analysis further confirmed 6G induced functional restoration and reactivation of p53 evident through the increased nuclear translocation of p53 in cervical cancer cells (Figure 2C). The 6G induced increase in the p53 and p21 protein levels was confirmed by immunobloting at 24h of treatment. As proteasome inhibitors are reported to increases p53 levels in cervical cancer cells [29], a concentration of 10nM Bortezomib, a known proteasome inhibitor, was used as positive control for p53 activation. Increase in the p53 levels in cervical cancer cells were comparable as that of 10nM Bortezomib (Figure 2D). Collectively, these results clearly suggested that 6G reactivates p53 and in turn increases p21 levels independently of the E6 transcriptional suppression in cervical cancer cells.

### 6G reactivates p53 *via* proteasome inhibition

**Figure 3:**
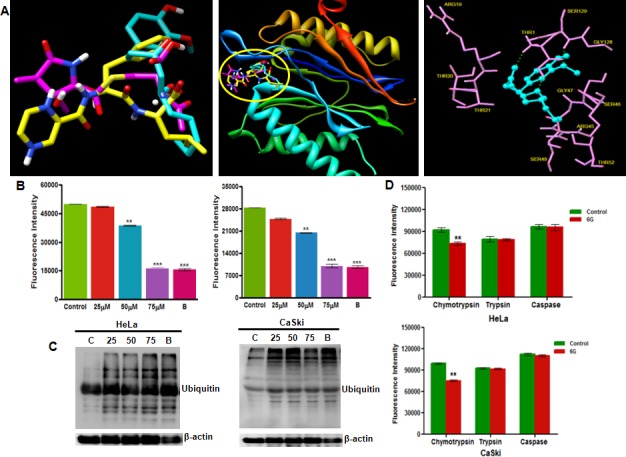
6G inhibits proteasome activity in HPV positive cervical cancer cells **A.** Molecular docking simulation of 6G showing its potential binding site in the proteasome subunit. (left panel) 3D structure comparison of 6G (blue) with Bortezomib (yellow) and Lactacystin (pink). (middle panel) Ribbon structure of beta-5 catalytic subunit of 20S core unit of proteasome. Similarity in the binding pockets of 6G, Bortezomib and Lactacystin for beta-5 subunit (encircled in yellow) responsible for chymotrypsin activity is indicated (right panel). 6G with interacting residues of beta-5 subunit. **B.** Proteasome activity of isolated proteasome was determined by Proteasome activity assay kit. Proteasomes from HeLa and CaSki cells were isolated manually after treatment with 6G (25, 50 and 75 μM) for 24h. Treatment with Bortezomib (10 nM) was done for positive control. Data are mean ± SD where **p < 0.01 and ***p < 0.001. Results presented are bar graphs of three independent experiments. **C.** Cells were lysed and subjected to western blotting for monitoring ubiquitinated proteins after treatment with 6G (25, 50 and 75 μM) and Bortezomib (10 nM). β-actin served as loading control. **D.** Proteasome activity in cultured cells was evaluated by incubating 6G treated (50 μM) HeLa and CaSki cells with specific substrates and measuring absorbance after 1h for specific cleavage. Significant decrease in chymotrypsin activity of cultured cells treated with 6G was observed in comparison to control cells. (**p < 0.01). The representative data of three independent experiments is presented as Mean ± SD.

HPV infection in cervical cancer cells maintain the endogenous p53 at negligible levels through its rapid proteasomal degradation by E6 and E6-AP proteins [9]. Therefore, the reactivation of p53 in these cells is achieved either through the suppression of E6 protein at transcriptional and translational levels [30, 31] or through the inhibition of proteasome activity by proteasome inhibitors thereby indirectly restoring p53 levels and activity [29]. Our results showed that 6G did not influence the E6 and E7 mRNA levels (Figure 2A) but instead increased the p53 and its target p21 expression comparable to that of the standard proteasome inhibitor Bortezomib (Figure 2B-2D), indicating the proteasome inhibitory activity of 6G in these cells. To confirm these findings, we performed molecular docking to explore the interactions between 6G and the proteasomal catalalytic β subunit. Our results showed that albeit the structural differences between 6G and the standard proteasome inhibitors Bortezomib and Lactacystin, it occupies the same binding pocket in the β-5 subunit (middle panel) of proteasome (Figure 3A) interacting with the similar set of binding residues as the other two (right panel) known to be responsible for its chymotrypsin activity [32]. Comparison of the binding energies further revealed that the affinity with which 6G interacts with beta-5 subunit was similar to that of Lactacystin but was lower than Bortezomib (Supplementary Table 1). To confirm the in silico prediction results, we employed biochemical assays to determine the effects of 6G treatment on isolated proteasome. Both HeLa and Caski cells were treated with 6G (25, 50 and 75 μM for 24h) and the proteasome activity was assessed. Bortezomib was used as a positive control (10nM). The results showed that, the 6G treatment decreased the activity of isolated proteasome in a concentration dependent manner in both the cell types at 24h (Figure 3B).

Since inhibition of proteasome is accompanied by an increased levels of ubiquitnated proteins, we next evaluated the levels of ubiquitinated proteins in 6G treated cells. We observed increased accumulation of ubiquitinated proteins in 6G treated cells similar to that of the bortezomib treatment (Figure 3C). We further explored the effect of 6G treatment on the three distinct activities, (*i.e.* chymotrypsin, trypsin and caspase like activities) essential for proteasomal function. 6G potently inhibited the chymotrypsin activity of proteasomal complex (Figure 3D). These results suggested that 6G inhibited proteasomal activity by binding to β-5 subunit of the proteasome core complex specific for chymotrypsin activity. Collectively these results confirmed 6G to be a novel inhibitor of chymotrypsin activity of proteasomal complex in the cervical cancer cells.

### 6G induces ROS generation leading to DNA damage and stabilization of p53

**Figure 4:**
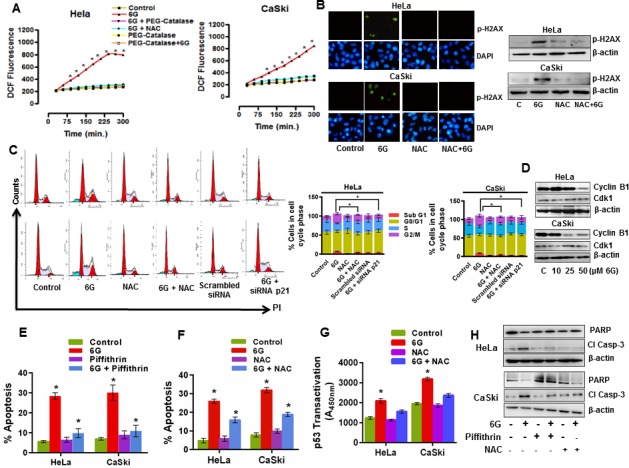
6G induced ROS and DNA damage enhances p53 dependent G2/M cell cycle arrest in cervical cancer cells **A.** Intracellular ROS generation was determined by CM-H2DCFDA staining based flourimetry. Cells were harvested, washed and stained for 30 min in the dark. The fluorescence intensity was measured by fluorimetry as slow kinetics until 6h after treatment with 6G. Significant increase in ROS levels were observed in 6G treatment group as compared to control and NAC (4 mM) and PEG-Catalase (200 IU) pre-treated groups (*p < 0.05). The data of three independent experiments is presented as Mean ± SD. **B.** Intracellular levels of p-H2AX (green) to measure cellular DNA damage was detected through immunofluorescence and western blotting in HeLa and CaSki cells. Cells were pre-treated with or without NAC for 1h and then treated with 6G (50 μM) for 12h. DAPI was used as counterstain for nucleus (blue) for immunofluorescence and β-actin served as loading control for western blot. The representative images of three independent experiments are presented **C.** Effect of 6G on cell cycle phase distribution was measured by flow cytometer. Histogram peaks and bar graph results indicated increase in percentage of cells in G2/M phase as compared to control groups (*p < 0.05) **D.** Western blot analysis of G2/M cell cycle phase transition related proteins after 6G treatment. **E.** Pre-treatment of cells with p53 inhibitor Piffithrin-α (10 μM) decreased percent apoptosis in HeLa and CaSki cells as detected by Annexin-V-FITC/PI staining. (*p < 0.05). **F.** and **G.** Pre-treatment of cells with NAC (4 mM) for significantly decreased percent apoptosis **F.** and p53 transactivation **G.** in HeLa and CaSki cells.*p < 0.05. **H.** Western blot analysis against cleaved Caspase-3 and PARP in HeLa and CaSki treated cells as indicated. β-actin served as loading control. The representative data of three independent experiments is presented as mean ± SD.

The therapeutic generation of reactive oxygen species (ROS) is a critical regulator of apoptosis in cancer cells [27]. Moreover, proteasome inhibitors also increase intracellular levels of ROS in cancer cells [33]. Therefore, we next examined the effects of 6G on ROS generation in cervical cancer cells. Time resolved fluorimetry for 6h indicated the increase in ROS generation in 6G (50 μM) treated cells which started as early as 2h of 6G treatment. Pre-treatment of cells with the ROS scavenger NAC (4 mM) reduced the ROS levels in cells comparable to that of control (Figure 4A). NAC is not a selective inhibitor of ROS and it is also reported to inhibit other off targets like mTOR [34], therefore we used another ROS scavenger PEG-Catalase (200 IU) to confirm the 6G mediated generation of ROS in both the cell lines. We observed that pretreatment of cells with NAC and PEG-Catalase exerted similar ROS scavenging effects on both the cells after 6G treatment (Figure 4A). The source of the 6G induced ROS generation in cervical cancer cells was attributed to the suppression of the MRC-I activity (Supplementary Figure S2). Increased levels of ROS in cancer cells bring about genotoxic stress through DNA damage [35]. Hence we further sought to identify whether 6G induces DNA damaging effects in cervical cancer cells. 6G treatment for 6h induced increased staining of p-H2AX in HeLa, and CaSki cells (Figure 4B, left panel). Immunoblotting results further confirmed the increased levels of p-H2AX after 6G treatment (Figure 4B, right panel). However, pre-treatment with the ROS scavenger NAC abrogated the expression of p-H2AX in both the cervical cancer cell lines. DNA damaging agents severely damage cellular DNA through different mechanisms including DNA oxidation. Severe and unrepairable damage to DNA halts replication at cell division and leads to cell cycle arrest at S or G2/M cell cycle phases [35]. Therefore changes in the cell cycle distribution pattern of HeLa and CaSki cells were studied post 6G (50μM) treatment at 24h. Significant accumulation of cells in the G2/M phase (*p < 0.05) was observed compared to that of controls (Figure 4C). ROS generation causes oxidative DNA damage in cervical cancer cells upon 6G treatment which resulted in G2/M arrest [22] and p21 is one of the major mediators of p53 dependent apoptosis in cancer cells [36]. Hence to explore the precise roles of ROS and p21 in 6G mediated G2/M arrest in cervical cancer cells, we used ROS scavenger NAC (4 mM) and p21siRNA. As presented in the Figure 4C, NAC pre-treatment partially reversed G2/M accumulation of cells in both the cell types, while genomic inhibition of p21 with targeted siRNA completely reversed G2/M accumulation of cells. p21, a cyclin dependent kinase inhibitor which binds and inhibit cyclin B1 and cdk-1 complex which is required for transition of G2 to M phase [37]. We further explored the levels of cell cycle proteins cyclin B1 and cdk-1, associated with the G2 to M phase cell cycle transition [37]. 6G treatment led to the marked reduction in the expression of cyclin B1 in both the cell lines in a dose dependent manner and time dependent manner (Figure 4D, Supplementary Figure S3). However no change in expression level of cdk-1 was observed upon 6G treatment. Collectively, these finding suggest that generation of intracellular ROS by 6G leads to apoptotic cell death in cervical cancer cells by inducing DNA damage and p53/p21 mediated G2/M cell cycle arrest. As the earlier results suggested 6G induced increase in the p53 level as well as ROS, we further sought to delineate the association between these key events in the 6G mediated pro-apoptotic effects. We pre-treated cervical cancer cells with the p53 inhibitor, Piffithrin-α (10 μM), and ROS scavenger, NAC (4 mM). Cells pre-treated for 1h with Piffithrin-α followed by 6G treatment (50 μM) for 24h completely attenuated 6G induced apoptosis (Figure 4E). On the contrary, NAC pretreatment only partially inhibited 6G mediated apoptosis in HeLa and Caski cells as compared to Piffithrin-α pre-treated cells (Figure 4F). P^53^ activation in response to oxidative stress and DNA damage is well documented [38, 39]. So, we next attempted to evaluate the role of ROS induced DNA damage in p53 transcriptional activation in these cells. HeLa and Caski cells were pre-treated with NAC followed by 6G treatment (for 18h) and p53 reactivation was quantified using the transactivation assay. Pre-treatment of NAC attenuated transactivation of p53 in HeLa and CaSki cells (Figure 4G). Western blotting analysis of the cleaved Caspase-3 and PARP also indicated that cells treated with 6G increased cleaved Caspase-3 and decreased PARP expression which was partially reversed by NAC pre-treatment and completely reversed by Piffithrin-α pre-treatment (Figure 4H). These results confirmed that the 6G induced p53 reactivation and ROS generation are intricately associated events similar to that of other natural compounds [40, 41]. Collectively, these results suggested that the high levels of ROS possibly maintained the p53 in the activated state [42] for induction of the pro-apoptotic effects.

### 6G potentiates anti-proliferative effects of cisplatin

**Figure 5:**
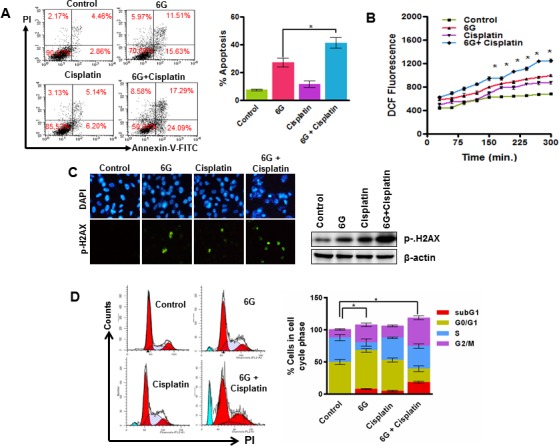
6G potentiates cisplatin induced cytotoxicity in HeLa cells **A.** HeLa cells were treated with 6G (50 μM) and cisplatin (2.5 μM) alone and in combination for 24h and analyzed for percent apoptotic cells. Results indicated significant increase in apoptotic cells in both the early and late apoptotic phase as compared to the control. *p < 0.05. **B.** Increase in ROS generation was measured as mean CM-H2DCFDA fluorescence in cells treated with combination of 6G and Cisplatin. **C.** Immunofluorescence of p-H2AX in cells after combined treatment of 6G and Cisplatin as indicated by fluorescence microscopy and western blotting in HeLa cells. **D.** Cells treated with 6G, Cisplatin or both were fixed, stained with PI and analyzed by flow cytometer for distribution of cell in each cycle phase. Bar graph indicates increased accumulation of cells in combination treatment with respect to individually treated and control groups *p < 0.05. The representative data of three independent experiments is presented as mean ± SD.

Cisplatin is used as a standard chemotherapeutic drug against a variety of cancers including cervical cancer. However, it is effective at high doses and cause severe side effects [7, 43]. Numerous research investigations have revealed that combination treatment with natural agents sensitize HeLa cells to lower doses of cisplatin [41]. Hence we next examined the previously unexplored property of 6G that whether it could chemosensitize HeLa cells to cisplatin. HeLa cells were treated with different concentrations of 6G in combination with sub IC50 doses of cisplatin for 24 h. Results indicated that 50 μM concentration of 6G sensitizes cervical cancer cells to 2.5 μM cisplatin. This combination increased percentage of apoptotic cells after 24h of treatment (Figure 5A). The quantification of the ROS levels suggested that the treatment of 6G (50 μM) and cisplatin (2.5 μM) in combination significantly increased the ROS levels in cervical cancer HeLa cells compared to either of the treatments alone (Figure 5B) indicating increased oxidative stress. Furthermore, the combination increased levels of p-H2AX, confirming induction of increased DNA damage (Figure 5C). Finally the cell cycle analysis indicated that the 6G + cisplatin combination induced a significant accumulation of cells in the G2/M cell cycle phase compared to that of either agents alone (Figure 5D). Furthermore, the *in vivo* xenograft experiments also showed decrease in the proliferation marker Ki67 and increase in the apoptotic TUNEL positive cells treated with the 6G + cisplatin combination compared to the individual treatment of either agents alone (Supplementary Figure S4A). Collectively, these results confirmed that 6G potentiates the anti-proliferative effects of cisplatin through induction of oxidative stress mediated DNA damage and cell death in cervical cancer cells.

### 6G inhibits proliferation of primary cervical cancer cells and tumor growth in HeLa xenografts

**Figure 6:**
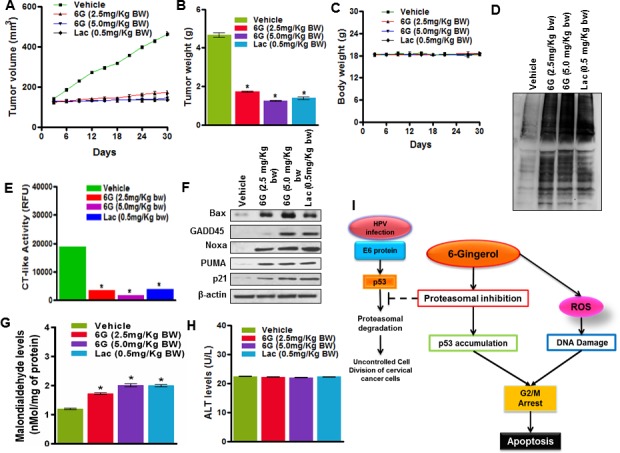
6G inhibits tumor growth in a HeLa xenografts model **A.** Tumor volumes of xenografted mice treated with or without 6G (2.5mg/Kg bw and 5.0mg/Kg bw) and Lactacystin (0.5mg/Kg bw). **B.** Average tumor weights of experimental mice. *p < 0.05 compared with healthy control group. **C.** Body weights of xenografted mice of indicated treatment groups. **D.** Increase in accumulation of ubiquitinated proteins in Xenograft tissue was determined by western blot analysis. **E.** Chymotrypsin (CT) like activity in xenograft tumor tissue was assessed within the indicated groups. **F.** Western blots of p53 target proteins Bax, GADD45, Noxa, PUMA and p21 in lysed tumor tissue isolated from xenografted mice. **G.** Malondildehyde levels in xenografts tumor cells **H.** ALT levels measured from plasma of xenografted mice with the indicated treatment groups. The representative data of three independent experiments is presented as mean ± SD. **I.** Schematic representation of molecular events triggered by 6G treatment in HPV infected cervical cancer cells.

To explore the effects of 6G on primary human cervical cancer cell proliferation, we cultured primary cervical cancer cells alone or with 50μM of 6G. Similar to the *in vitro* results, 6G significantly inhibited the proliferation and the chymotrypsin activity of the primary cervical cancer cells (Supplementary Figure S4B and C). We further sought to validate the anti-tumor effect of 6G *in vivo* using a HeLa xenograft model. Nude mice were inoculated with three million cultured HeLa cells subcutaneously and tumor growths were monitored. On the onset of significant tumor growth mice were administered with 6G (2.5mg/Kg and 5.0mg/Kg body weight) and for positive control Lactacystin (0.5mg/Kg body weight) for 45 days. Significant (*p < 0.05) decrease in tumor volume and tumor weight was observed in tumor bearing mice treated with 6G at both the doses and Lactacystin (Figure 6A and 6B). There was no significant differences between the body weights (Figure 6C) or the hematological / biochemical and osteogenic parameters between of the experimental mice groups treated with the aforementioned or higher doses of 6G (Supplementary Tables 3 & 4, Supplementary Figure S5). The proteasome inhibitory activity of 6G was further validated through assessment of ubiquitinated proteins and chymotrypsin like activity in the mice xenograft tissues. The results indicated increased accumulation of ubiquitinated proteins and inhibition of the chymotrypsin activity (Figure 6D and 6E). In addition, immunohistochemical staining of xenograft tumor tissue sections also showed increased staining of p53 protein in 6G and Lactacystin treated groups as compared to untreated control tissue (Supplementary Figure S4D). To draw consistency with the *in vitro* results changes in protein expression of the p53 target genes were also monitored. Protein levels of Bax, GADD45, Noxa, Puma and p21 were significantly reduced in treated groups confirming the *in vitro* p53 reactivation (Figure 6F). To confirm the generation of ROS *in vivo*, in the tumor xenograft tissues, expression of the oxidative stress biomarker malondialdehyde (MDA) was checked which was found to be elevated in the treated groups (Figure 6G). To test whether administration of 6G created a safe pharmacokinetic profile, generation of alanine transaminase (ALT) enzyme was measured (Supplementary materials and Methods) in the plasma of treated mice. The results indicated no elevation in the ALT levels, confirming the administration of 6G to be safe in *in vivo* (Figure 6H). Collectively, our *in vivo* results were in agreement with our *in vitro* studies validating the 6G induced p53 reactivation though proteasomal inhibition in cervical cancer cells (Figure 6I) along with its lack of toxicity in the experimental animal model.

## DISCUSSION

The natural polyphenolic compound 6G has been shown to induce apoptosis in a variety of human cancer cells through several mechanisms, thereby exhibiting the potential as a chemotherapeutic agent [20]. Our recent study also demonstrated the chemopreventive effect of 6G mediated through the oxidative stress induced DNA damage and activation of the miR27b expression in myeloid leukemia cells with apoptotic induction [27]. As the inactivation of p53 functions is a universal feature of cervical cancer cells, small molecule induced reactivation of p53 and its associated apoptotic signaling pathways is a promising strategy for cancer therapy [44]. In this study, we provide experimental evidence that 6G treatment inhibits proliferation of HPV-positive cervical cancer cells through proteasome inhibition mediated p53 reactivation, increase of oxidative stress, induction of DNA damage associated G2/M cell cycle arrest and apoptosis.

Dietary polyphenols are known to inhibit the proliferation of HPV-immortalized and HPV-positive cancer cells, through induction of apoptosis, growth arrest, inhibition of DNA synthesis and modulation of signal transduction pathways acting at several steps in the cascade of cell transformation [45]. Our preliminary results exhibited the potent anti-proliferative effect of 6G (50μM) in HPV positive cervical cancer cell lines in agreement with earlier studies underscoring the therapeutic potential of dietary polyphenols in the prevention and therapy of cervical cancer [45].

In HPV positive cervical cancers, the viral oncoproteins E6 and E7 induce transformation by targeting the tumor-suppressor p53 for ubiquitin-dependent proteasomal degradation resulting in reduction of its levels aiding cancer progression [28]. Therefore, reactivation of p53 is a potential therapeutic approach for treatment of cervical cancers expressing wild-type p53. Stabilization of p53 by prevention of its degradation could recover sufficient levels of wild-type p53 to trigger apoptotic cell death in cancer cells. Transcriptional inhibition of HPV oncoproteins is known to reactivate the tumor suppressor p53 in cervical carcinoma cells [30]. However, we found that 6G induced p53 reactivation did not involve HPV oncoprotein inhibition leading us to explore its effect on proteasome activity as cervical cancer cells exhibit an increased requirement for ubiquitin-dependent protein degradation [15]. We found for the first time, that the beta 5 subunit of the proteasome is one of the targets of 6G *in vitro*, inhibition of which could reactivate p53 by blocking the actions of proteasome, thus preventing p53 from being degraded [15]. Our results showed that the 6G treatment led to the dose-dependent accumulation/stabilization of p53. Stabilization of the levels of p53 was observed concomitantly with accumulation of high-molecular weight poly-ubiquitinated species as early as 2 hours post-treatment, well before induction of cell death (data not shown). Hence this data confirmed that the stabilization of p53 is the cause rather then the consequence of the decrease in cell viability in cervical cancer cells.

ROS generation in cancer cells is attributed to the regulation of iNOS, Nox NADPH oxidases, cytochrome p450, down regulation of antioxidative proteins or inhibition of MRC [27]. Our results consistent with our earlier study [27], showed that 6G induced inhibition of MRC I activity and led to the ROS production (Supplementary Figure S3). 6G induced ROS accumulation is known to induce multiple signaling pathways [20, 27]. In the present study we observed 6G induced DNA damage characterized by increased p-H2AX staining was the result of increased ROS production in both the cells [23, 26] since pre-treatment of the ROS scavenger NAC reduced p-H2AX staining and protein expression in these cells. These effects of 6G observed in this study is in agreement with the naturally occurring polyphenols with documented effects on ROS production, cell-cycle regulation and induction of apoptosis [20, 26, 46]. We also observed that either inhibition of ROS or the genomic inhibition of p21 reversed 6G induced G2/M cell cycle arrest in both the cells. However 6G induced apoptosis was completely reversed by pre-treatment with p53 inhibitor, piffithrin in both the cells while NAC pre-treatment only partly reversed 6G induced effects. Notably 6G induced p53 transactivation was also inhibited by NAC pretreatment. Previous studies have shown that increased intracellular ROS is known to stabilize p53 [42]. Hence it is plausible from this data that 6G induced proteasome inhibition and oxidative stress through increased ROS stabilized p53 and its downstream targets such as p21 in response to DNA damage [37] resulting in halting of cell cycle progression leading to apoptosis. These results are in accordance with the previous experimental findings where it has been shown that proteasome inhibition and p53 dependent apoptosis are two independent phenomena of proteasome inhibitors [47-48]. However our results confirmed p53 reactivation and apoptosis through proteasomal inhibition to be more prominent effect of 6G in HPV positive cells while generation of ROS as a supporting mechanism in these cells. These results are supported by many studies in which natural molecules generate ROS as a boosting mechanism in cancer cell death [49-50].

ROS toxicity induced by certain chemotherapeutic agents can be an effective means of eradicating malignant cells, it is useful to consider effective ways of achieving significant synergy through combination of agents with similar ability to alter redox conditions. Cisplatin, often included in cervical chemotherapy regimens, has several mechanisms of action; one of which is the formation of free radicals leading to oxidative stress [51]. 6G potentiated the toxic effect of cisplatin by amplifying ROS generation. The ability of 6G to enhance intracellular accumulation of cisplatin by reducing its efflux agrees with previous studies that natural agents reverse multidrug resistance involved in regulating the efflux of chemotherapeutic drugs from cells [50-52]. It is possible that 6G exacerbated cisplatin-induced apoptotic cell death in cervical cancer cells by coupling enhanced oxidative stress with increased accumulation of cisplatin. This increased efficacy of 6G in inducing apoptosis of cervical cancer cells through disruption of cellular redox balance, in conjunction with other ROS-generating anticancer therapeutic modalities, suggests that a strategy combining these agents warrants further investigation in clinical trials.

Finally, based on the high antiproliferative efficacy of 6G against cervical cancer cells in vitro, we tested its efficacy against Hela subcutaneous xenografts in athymic nude mice. In this study, the animals treated with 6G (2.5 and 5.0 mg/kg bw) for 6 weeks exhibited a significant reduction in tumor volume (nearly 65%). Consistent with the *in vitro* findings, we observed proteasomal inhibition and increase in p53 levels in the 6G-treated mice xenografts. Expression of the cell cycle regulators and other apoptotic markers were also observed consistent with the *in vitro* studies. More importantly, our work also demonstrates that inhibition of the tumor proteasomal chymotrypsin subunit is an *in vivo* biological target of 6G in tumor tissues, associated with tumor growth inhibition [28]. The potent anti-proliferative effect of 6G *in vivo* is mediated through proteosomal inhibition and p53-reactivation (Supplementary Figure S4D) leading to inhibition of proliferation and induction of apoptotic cell death.

The speculated bone loss inducing effects in the female [53] and relatively high effective doses of 6G against multiple cancer types is perceived as major road blocks in its clinical translation. However, our earlier study [27] and a recent study has provided experimental evidence for 6G as an anti-cancer, anti-inflammatory and anti-osteoporetic agent [54] with favorable toxicity, pharmacokinetics and pharmacodynamics profiles at relatively higher doses (≈50μM). Moreover, our results with the treatment of 6G (at doses of 2.5, 5.0 and 50 mg/kg BW) for 4 weeks in the female mice did not induce any systemic toxicity (Supplementary Table 3) or bone specific detrimental effects (Supplementary Table 4 and Supplementary Figure S5). Similarly, clinical trials with 6G in healthy human subjects have established the favorable of toxicity, pharmacokinetic and pharmacodynamics profiles at high doses [55]. Furthermore, the HED (Human Equivalent Dose) calculation of 6G according to the BSA (Body surface area) method [56] based on preclinical models for an average (60Kg) weight person ranges between 30mg to 123.3mg/Kg body weight, compared to the does used for the *in vivo* experiments in this study (doses 2.5mg/Kg BW to 10mg/Kg BW used in experimental mice). The proteasome inhibitory activity of 6G at relatively higher doses of 6G is in agreement with other polyphenolic natural compounds [55-58]. Furthermore, studies have suggested that higher consumption and systemic concentration of polyphenols reduce risk of cancer and is safe due to their rapid metabolism and excretion [59-61]. However, future studies on development of nano particle based delivery systems for 6G can significantly reduce its effective therapeutic doses like the other natural anticancer agents [62-63]. Similarly, based on our present findings the potential of 6G should be evaluated as a chemosensitizer in future studies / clinical trials.

In conclusion, our results demonstrate that the natural compound 6G is a potent inducer of p53 reactivation in HPV positive cervical cancer cells through proteasome inhibition, underscoring its translational relevance as a chemotherapeutic agent as well as its potential as a chemosensitizer for conventional chemotherapeutic drugs like cisplatin. This study supports the need for further studies with 6G as a potential therapeutic agent, particularly for cervical cancers with aggressive phenotypes.

## MATERIALS AND METHODS

### 6G extraction and purification

6G was extracted and purified in the Medicinal and Process Chemistry Division (CSIR-Central Drug Research Institute, India) from rhizomes of ginger (Zingiber officinale) as described previously [27]. The purified compound was dissolved in dimethyl sulphoxide (DMSO) (Sigma, St. Louis, MO, USA) to a final concentration of 100 mM, aliquoted and stored in −20°C until further use.

### Cell culture, transfections and reagents

Cervical cancer cells HeLa, CaSki and SiHa were obtained from American Type Culture Collection (ATCC; Manassas, VA, USA) and maintained in DMEM, RPMI-1640 and MEM respectively, supplemented with 10% fetal bovine serum, 1% antibiotic and antimycotic solution with 5% CO_2_. For transfections cells (4 × 10^4^) were seeded in 6 well plates and transfected with p21 siRNA (Ambion, Austin, TX, USA) to a final concentration of 25nmol/L, using Lipofectamine (Invitrogen, Carlsbad, CA, USA), media was changed after 4 h. Twenty-four hours after transfection, cells were treated with 50μM of 6G for 24 h. Cells were collected for western blotting analysis or fixed in 70% ethanol for flow cytometry studies. Bortezomib, Lactacystin, piffithrin, MTT, were purchased from Sigma-Aldrich (St. Louis, MO, USA). Apoptosis detection kit was obtained from Invitrogen (Carlsbad, CA, USA). Proteasome activity assay kit was purchased from Millipore (Billerica, CA, USA), Proteasome substrates II, III and IV were obtained from Calbiochem (Merck, Darmstadt, Germany). p21 siRNA was purchased from Ambion (Austin, TX, USA). Antibodies against p-H2AX (J0510), p53 (sc-6243), β-actin (K0211), were from Santa Cruz Biotechnologies (Santa Cruz, CA, USA), p21(2947), Ubiquitin (3936), Cleaved Caspase-3 (9664), Bax (2774), Noxa (14766), PUMA (12450), Ki-67 (9449) from Cell Signaling Technology (Beverly, MA, USA), PARP (ab32138) and GADD45 (ab180768) from Abcam (Cambridge, UK), Cyclin B1 (610219), Cdk 1(610037) from BD Pharmingen (San Diego, CA, USA) and p53 (Dako, Carpinteria, CA, USA).

### Cell viability

Cells were seeded in 96 well plates at a density of 3000 cells per well and incubated at 37°C for 12h. Afterwards, culture media was replaced and cells were treated with different concentration of 6G 25-200 μM for 24, 48 and 72h. After the respective time point cells were again incubated with MTT (5mg/ml) for another 2h, formazons so formed was dissolved in DMSO and absorbance was taken at 540nm and % cell viability was calculated.

### Flow cytometry

Detection of apoptosis and cell cycle arrest was performed by flow cytometry as described previously [27]. Briefly, cellular apoptosis detection was done through Annexin-V-FITC and propidium iodide (PI) staining. Control and treated cells were stained and cells and analyzed through flow cytometer (FACS Calibur, BD Biosciences, San Jose, California, USA). For cell cycle analysis, cells were seeded starved in serum free media for 6 h at 37°C, followed by treatment with 6G for 24h. Cells were harvested with tyrpsin, washed with PBS and fixed in 70% ethanol for overnight. Fixed cells were washed with PBS and stained in PI solution with PI (50μg/ml) and RNAseA (50μg/ml) in PBS and incubated for 30 min at room temperature. Samples were acquired with FACS and analyzed with Modfit Software.

### RNA isolation and real time PCR

RNA from control and treated cells were isolated by Trizol method and cDNA synthesis was done with 1μg of total RNA using First Strand cDNA synthesis kit real time PCR was done by using Syber green dye according to standardized protocol [27] for E6, E7 and p21 gene with 18S as endogenous control.

### p53 transactivation assay

p53 transactivation was assessed by p53 transactivation assay ELISA kit (Active Motif, Carlsbad, CA, USA) by using nuclear extracts (Nuclear and Cytosolic Extraction Kit, Pierce, Rockford, IL, USA) isolated from cells treated with 6G according to manufacturer's instructions.

### Immunocytochemistry and confocal microscopy

Cells (1 × 10^3^) were seeded on pre-sterilized circular coverslips and treated with 6G for 12h. After 12h both treated and untreated cells were fixed with 4% paraformaldehyde for 15mins at room temperature, washed twice with PBS and permeabilized in 0.3% TritonX-100 in PBS for 5 min. Blocking was done in 5% BSA for 1h at RT followed by incubation with primary antibodies p53, p21 and p-H2AX overnight at 4°C. Cells were washed and incubated with Alexa Fluor-488 conjugated secondary antibody (Molecular Probes) for 1h at RT, and counter stained with DAPI. Images were analyzed by confocal microscope (Carl Zeiss, Germany) under 63x magnification.

### Western blot analysis

Western blotting was done as described elsewhere [27]. Briefly, cell were lysed in RIPA lysis buffer containing protease and phosphatase inhibitors (EMD Biosciences, Germany) and total protein was estimated with Bradford reagent. 40μg of protein was separated on 10-12%SDS-PAGE and transferred on nitrocellulose membrane (Millipore, Billerica, CA, USA). The membranes were blocked for 1h at RT and incubated with primary antibodies p-H2AX, β-actin, p21, cdk1, cyclin-B1, p53 (1:2000) followed by suitable HRP conjugated secondary antibodies (1:5000). Blots were developed using the ECL Detection System (Millipore).

### *In silico* analysis

Molecular docking was performed with Autodock software version 4.2. The 3D structure of Proteasome was downloaded from Protein Data Bank (PDB) and of 6G from PubChem Id CID 442793. Docking simulations were performed with LamarkianGenetic Algorithm (LGA). Each docking experiment was performed for 100 runs containing population size of 300 individuals and it was terminated with a maximum number of 2500000 energy evaluations or a maximum number of 27000 generations. Further for each run the top ranked individual in the population was set to survive into the next generation. Mutation and crossover rates were set at 0.02 and 0.80 respectively. Visualization was performed using UCSF Chimera1.6.2rc.

### Proteasome activity assay

Isolated 26S proteasome activity was evaluated using Proteasome activity Assay Kit according to manufacturer's instructions. Briefly, cells were lysed in (50 mM HEPES (pH 7.5), 5 mM EDTA, 150 mMNaCl and 1% Triton X-100) for 30 min. on ice with regular vortexing for 15 secs after every 10 min of incubation and centrifuged for 15 min at 4°C at 14,000 × g to isolate proteasome. 10μg of isolated proteasome was incubated with fluorogenic substrate in 1x assay buffer provided with the kit for 1h in dark at 37°C. Fluorescence intensity was measured by fluorimeter (BMG fluostar Omega, BMG Technologies, Offenburg, Germany) with a 380/460 nm filter set. Proteasome activity assessment in live cells was determined by using specific substrate. Cells were treated with or without 50 μM 6G for 24h. Both treated and untreated cells were harvested by trypsinization and washed with serum free media. Cells were resuspended in serum free media containing Proteasome substrates II, III or IV and incubated for 1h in dark at 37°C. Cells were pellet down and resuspended in serum free media and fluorescence was measured at 380/460 nm.

### ROS estimation

Gingerol induced ROS generation was evaluated by staining cells with CM-H2-DCFDA dye (Invitrogen, Carlsbad, CA, USA). Cells were harvested by trypsinization, pretreated with or without NAC (4 mM) at 37°C for 1 h and washed with PBS twice. Thereafter cells were resuspended in serum free media and stained with 5 mM CM-H2-DCFDA in dark with continuous shaking for 30 min. Stained cells were washed and resuspended in serum free media. Both NAC pretreated and untreated cells were treated with 6-gingerol and fluorescence was measured in slow kinetics mode for 6 h in BMG Fluostar Omega spectrofluorimeter.

### Primary human cervical cancer cell culture

Samples were collected at the time of diagnosis from twelve patients diagnosed with invasive cervical cancer (5 squamous cell carcinoma and 7 adenocarcinoma samples, international federation of gynecology and obstetrics (FIGO) stages IIB, Supplementary Table 2) and six healthy donors (Control samples), enrolled for treatment at the department of obstetrics and gynecology of the King George Medical College, Lucknow using a research protocol approved by our Institutional review board (CDRI1054). Informed consent was obtained according to the declaration of Helsinki. Immediately after surgery, fresh cervical tissue samples were collected in cold phosphate buffered saline (PBS) and washed thrice with PBS. Subsequently, the small pieces of minced tissue were incubated in phenol-red free DMEM/F12 (30 ml) containing type I collagenase and DNase I for 3∼5 hr at 37°C in a shaker incubator. After the incubation period the cell suspension was filtrated through a 100 μm nylon cell strainer (BD) for thrice and a 70 μm nylon cell strainer for once. After filtration, cells remaining in the filtrate were collected by centrifugation at 1500 rpm for 5 min and washed once with PBS. Finally the primary cells were re-suspended in phenol-red free DMEM/F12, and plated into 100 mm^2^ dishes and the indicated treatments were carried out.

### *In vivo* HeLa xenograft assay

All the animal experiments were performed in accordance with the Institutional Animal Care and Use Committee procedures and guidelines of the CSIR-Central Drug Research Institute. Male (nu/nu) nude mice (5 to 6 weeks old) were maintained in pathogen-free conditions. Exponentially growing HeLa cells (3×10^6^) were injected subcutaneously in each mice for tumor induction. Once the mice attained a tumor volume of 90 to 100 mm^3^, the mice were divided into various groups as per the indicated treatments of six (*n* = 6) mice each. Treatments of the vehicle, 6G (2mg/kg bodyweight), 6G (5mg/kg bodyweight) and Lac (0.5mg/kg bodyweight) were administered through the intraperitoneal route on every alternate day till 45 days. Kinetics of tumor formation was estimated by measuring tumor size and volume at 3-day intervals. Tumor size was measured with calipers, and tumor volume was determined using the formula: volume = 0.5 × width^2^ × length. At the end of the experiment, animals were sacrificed through cervical dislocation and tumors were dissected, weighed and used for further analysis. The lipid peroxidation was quantified using the fluorometric Lipid Peroxidation (MDA) Assay Kit (Abcam, ab118970).

### Statistical analysis

Statistical analysis was performed using Graphpad Prism 4.0 software (Graphpad Softwares Inc., La Jolla, CA, USA). Comparison of multiple columns data sets were analyzed by one way ANOVA using Newman-Keuls comparison test. Paired comparisons of the data were conducted using a paired *t* test, and all data are presented at mean values ± S.D. Differences were considered significant at a *p* < 0.05 level of confidence.

## SUPPLEMENTARY MATERIAL FIGURES AND TABLES


