# Supplementary material for: Proteasome inhibition mediates p53 reactivation and anti-cancer activity of 6-Gingerol in cervical cancer cells
Source: Oncotarget. 2015 Nov 25;6(41):43310–25. doi: 10.18632/oncotarget.6383 (PMC4791234; doi:10.18632/oncotarget.6383)
Supplement: Supplementary file 1 [file oncotarget-06-43310-s001.pdf]

Proteasome inhibition mediates p53 reactivation and anti-cancer activity of 6-Gingerol in cervical cancer cells

Supplementary Material

Analysis of Biochemical parameters

Heparinized blood samples (50µl /mouse) were obtained on day 0 and at the end of the treatment period by sub mandibular bleeding. The clinical chemistry of the blood including parameters for AST: aspartate aminotransferase; ALT: alanine aminotransferase; BUN: blood urea nitrogen; WBC: White blood cells; Hgb: hemoglobin and PLT: Platelets were obtained using the Hemagen Analyst^{VR} Benchtop Chemistry System (Hemagen Diagnostics, Inc. Columbia,USA).

Mitochondrial complex activity assay

The activity of MRC complexes was determined with Mitochondrial Respiratory Chain Complex-I Activity Assay Kit (Novagen, Merck, Darmstadt, Germany) as per the manufacturer's instructions.

Micro CT Analysis

Right femoral bones were harvested and analyzed for bone mineral content, cortical thickness, and cortical bone area by microCT (Skyscan 1172, Aartselaar, Belgium). The the length of each femoral bone was measured at mid-diaphysis, and 40 mid-diaphyseal slices were used for the anlysis. The data were collected at 55 kVp and 72 µA at a resolution of 12 µm, and volumetric analysis was performed using Skyscan software.

Primers used in the study:

Gene	Forward	Reverse
**E6**	GCAAATGCAGGTGTGGATAATA	TCTAATGGTGGACAATCACCTG
**E7**	CTCTGTGGCCCATTGTATCA	GCGAGTCACATAATCATCGGTA
**p21**	ATGAAATTCACCCCCTTTCC	CCCTAGGCTGTGCTCACTTC
**18S**	CTTGGATGTGGTAGCCGTTT	TAACGAGGATCCATTGGAGG
**ALP**	AACCCAGACACAAGCATTCC	GAGACATTTTCCCGTTCACC
**BMP2**	TGAGGATTAGCAGGTCTTTG	CACAACCATGTCCTGATAA
**SMAD7**	GCAGGCTGTCCAGATGCTGT	GATCCCCAGGCTCCAGAAGA
**LEF1**	CCCACACGGACAGTGACCTA	TGGGCTCCTGCTCCTTTCT
**PTH1R**	CCTAAACTCC- CACTGTCCTT	CCTCAGGTTCTTGATTCACT
**Sost**	TCCTCCTGAGAACAACCAGAC	TGTCAGGAAGCGGGTGTAGTG
**ATF4**	GCA AGG AGG ATG CCT TTT C	GTT TCC AGG TCA TCC ATT CG
**AXIN2**	AGTGAGACGCTCTCCCTCACCA	GAAACGGGCATAGGTTTGGTGGAC
**FoxO1**	CAAAGTACACATACGGCCAATCC	CGTAACTTGATTTGCTGTCCTGAA
**OPG**	TCA AAG GCA GGC GAT ACT	CAA TGT CTT CCT CCT CAC TGT
**GAPDH**	TGCTGAGTATGTCGTGGAGTCT	CATATTTCTCGTGGTTCACACC

Supplementary Table 1: Comparison of docking score, binding energy and interacting residues between 6G and standard inhibitors Bortezomib and Lactacystin.

S.No	Ligand	Receptor	H-Bonds	Interacting residues	Docking Score
1	Bortezomib	Proteasome	4 (GLY47, THR1, THR21, ALA49)	GLY47, THR20, ARG45, ARG19, THR1, THR21, THR52, THR22, ALA49, SER168, LYS33	-7.17
3	Lactacystin	Proteasome	4 (THR21,THR1, GLY23, THR21)	GLY23, GLY47, THR22, THR1,THR20, THR21, ALA49, SER48, SER46	-5.38
2	Gingerol	Proteasome	3 (THR1, SER129)	GLY128 , ARG19 , SER129, THR1, SER48, GLY47, THR20, SER46, THR52, ARG45, THR21	-4.94

Supplementary Table 2: Pathological characteristics of primary cervical carcinoma cells used in the study

Sample No	HPV Type	Histology	Stage (According to the FIGO System)	Site of Disease
1	16	Squamous Cell Carcinoma	IIB	Cervix
2	18	Adenocarcinoma	IIB	Cervix
3	16	Squamous Cell Carcinoma	IIB	Cervix
4	16	Squamous Cell Carcinoma	IIB	Cervix
5	18	Adenocarcinoma	IIB	Cervix
6	18	Adenocarcinoma	IIB	Cervix
7	18	Adenocarcinoma	IIB	Cervix
8	18	Adenocarcinoma	IIB	Cervix
9	18	Adenocarcinoma	IIB	Cervix
10	18	Adenocarcinoma	IIB	Cervix
11	16	Squamous Cell Carcinoma	IIB	Cervix
12	16	Squamous Cell Carcinoma	IIB	Cervix

Supplementary Table 3: Biochemical parameters of 6G from blood samples of experimental mice.

Groups	AST (U/L)	ALT (U/L)	BUN (mg/dL)	Hgb (mg/dL)	WBC (K/µl)	PLT (K/µl)
Control	117±9.1	39±2.1	57±2.1	12.2±0.3	2.9±0.2	841±33
6G (2.5 mg/Kg BW)	126±7.4	36±1.9	56±1.9	12.2±0.5	2.6±0.15	835±22
6G (5.0 mg/Kg BW)	118± 6.2	39±2.1	54±3.3	11.8 ±0.9	2.6±0.12	837±21

The representative data of three independent experiments is presented as mean ± SD.

Supplementary Table 4: Summary of microCT analysis of femoral bone.

Parameters	Control	6G (2.5mg/Kg BW)	6G (5mg/Kg BW)	6G (50mg/Kg BW)
**Cortical BMC (mg)**	0.566 ± 0.014	0.565 ± 0.018	0.567 ± 0.012	0.564 ± 0.013
**Cortical thickness (mm)**	0.118 ± 0.004	0.117± 0.002	0.119 ± 0.005	0.118 ± 0.003
**Cortical area (mm^2)**	0.952 ± 0.019	0.956 ± 0.023	0.957 ± 0.026	0.954 ± 0.025
**Trabecular BMC (mg)**	0.078 ± 0.012	0.081 ± 0.012	0.080 ± 0.012	0.079 ± 0.011
**Trabecular thickness (mm)**	0.068 ± 0.001	0.069 ± 0.008	0.065 ± 0.003	0.063 ± 0.002
**Trabecular number (1/mm)**	2.149 ± 0.094	2.149 ± 0.098	2.149 ± 0.102	2.147 ± 0.099

BMC = bone mineral content. The representative data of three independent experiments is presented as mean ± SD.

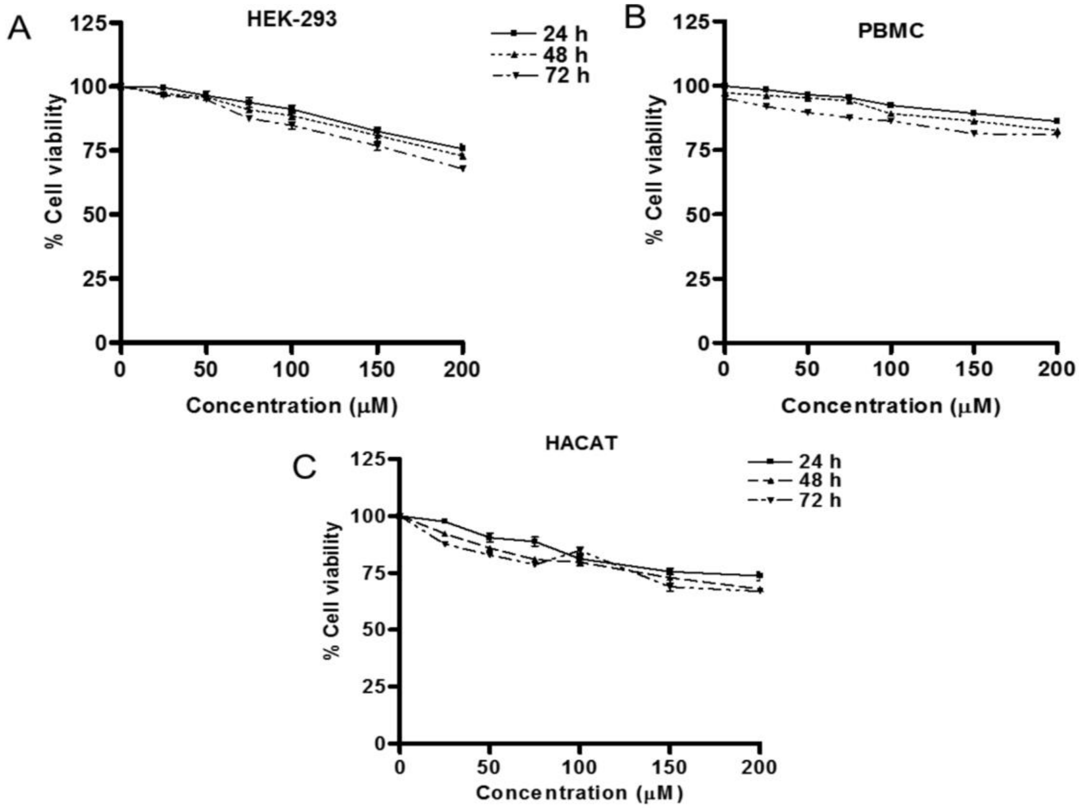

Figure S1. Safety profile of 6G on non-cancerous normal cell lines. 6G exhibited safer profiles with non-transformed normal cells. Non-transformed HEK-293 (A), Peripheral derived mononuclear cells (PBMC) (B) and HACAT (C) cells were treated with different concentrations of 6G for 24, 48 and 72h and subjected to MTT assay. The representative data of three independent experiments is presented as mean ± SD.

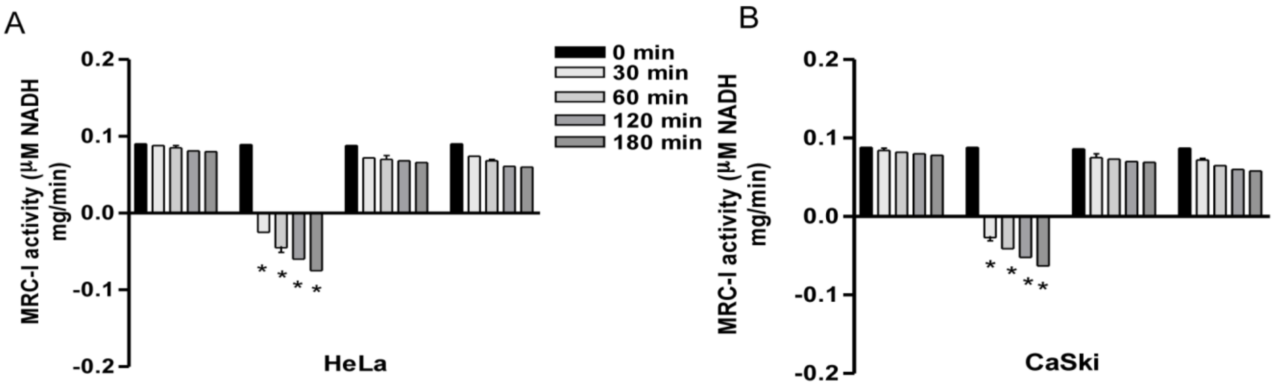

Figure S2. Effect of 6G on MRC-I. HeLa (A) and CaSki (B) cells were treated with 6G and MRC-I complex activity was determined for indicated time points. Reduction in MRC-I complex activity was observed in comparison to control *p≤ 0.05. The representative data of three independent experiments is presented as mean ± SD.

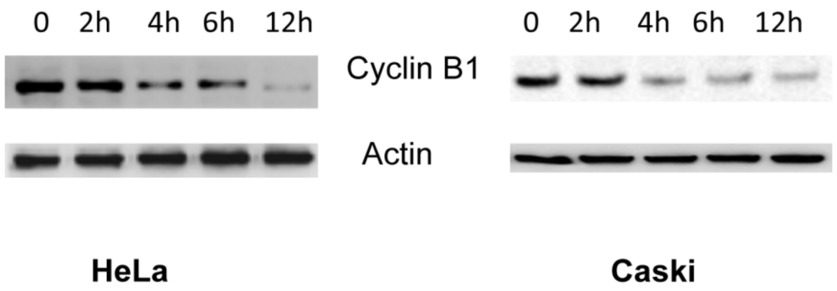

Supplementary Figure S3. Effect of 6G on Cyclin B1 expression in Cervical cancer cells. The cervical cancer cell lines (HeLa and Caski) were treated with 6G for the indicated time periods. The cell lysates were immunoblotted for cyclin B1 expression. The representative data of three independent experiments is presented.

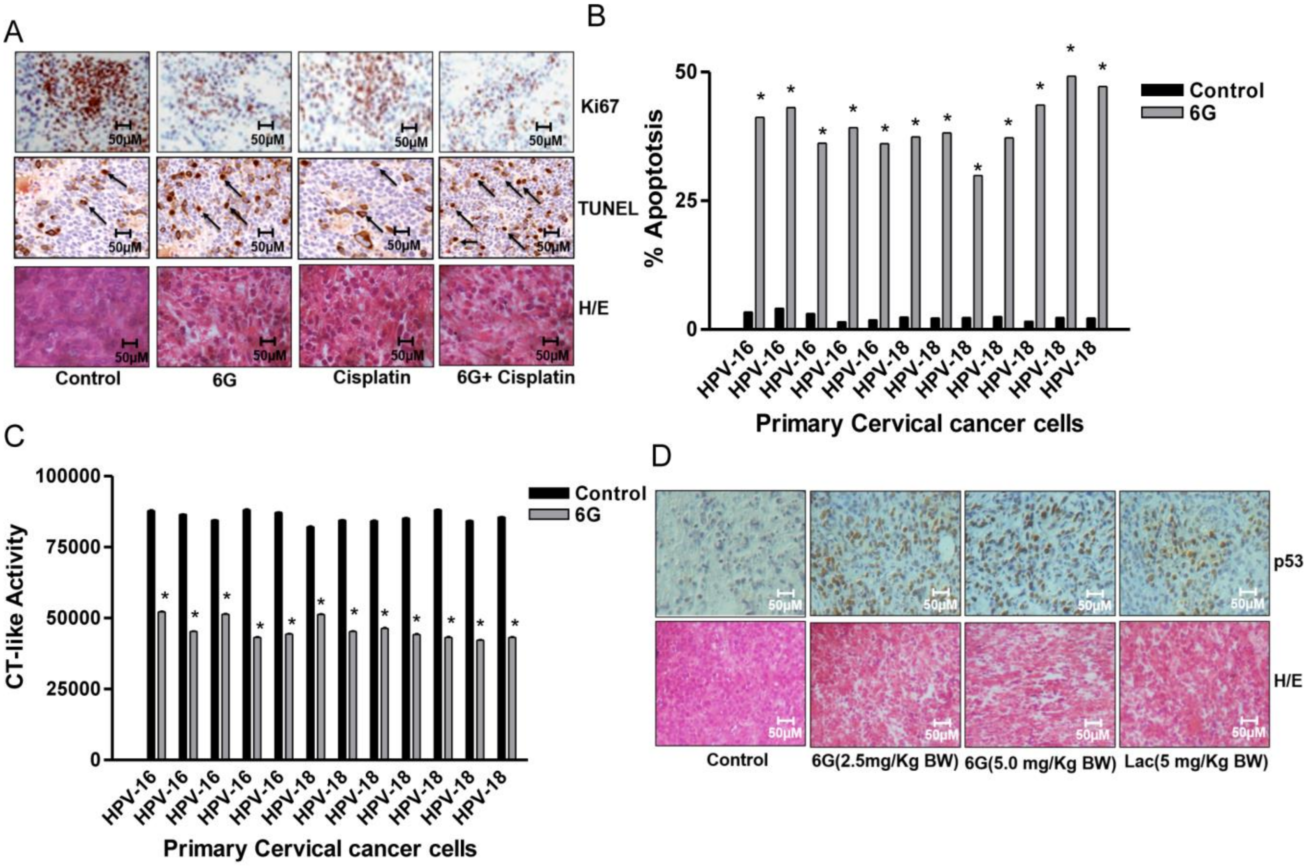

Figure S4. Effect of 6G induced apoptosis on primary cervical cancer cells and Xenograft model. (A) Tumor Xenograft sections were stained for Ki67 and TUNEL positive cells in Control, 6G, Cisplatin and 6G + Cisplatin treated groups. Arrows indicate TUNEL positive cells. (B) Cells derived from both HPV 16 and 18 positive cells were isolated, cultured and treated with 6G and percent apoptosis was determined by Annexin-V-FITC/PI staining. Bar graphs are representative of ±SD of three independent experiments in comparison to control group *p≤ 0.05. (C) Cells treated with 6G were subjected to Chymotrypsin (CT)- like activity assay. Relative fluorescence was measured in comparison to control *p≤0.05. The representative data of three independent experiments is presented as mean ± SD. (D) Immunohistochemical staining for p53 gene in tumor xenograft tissue sections in indicated groups.

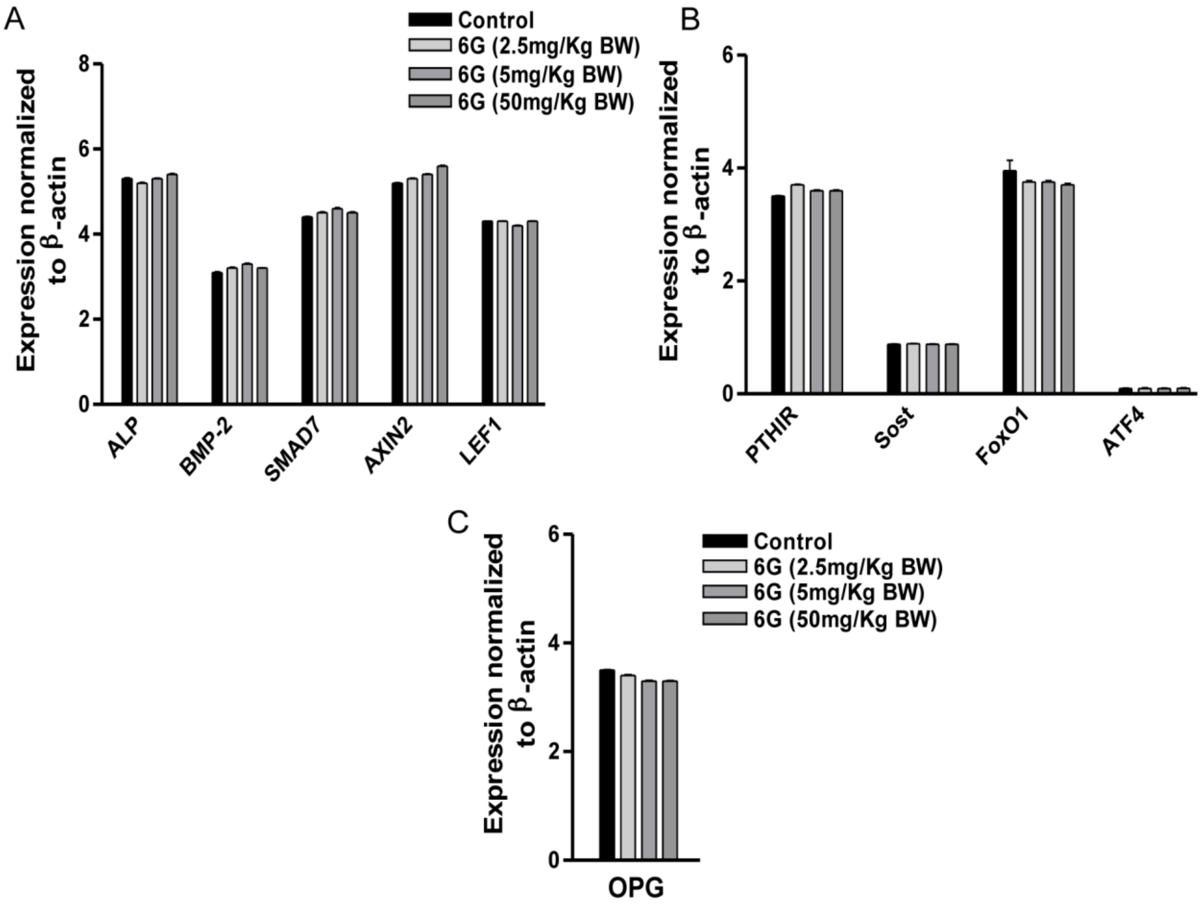

Figure S5. Effect of 6G on osteogenic parameters. Mice (N=8 per Group) were treated with control vehicle or or the indicated doses of 6G for 4 weeks. (A) Realtime RT-qPCR analysis of RNA isolated from calvarial bone for osteoblastic differentiation markers, (A) alkaline phosphatase (ALP), (B) BMP-2 and its target gene, Smad7, and Wnt target genes, Axin-2 and LEF-1. (B) Realtime RT-qPCR analysis of RNA isolated from femoral cortical bone, devoid of bone marrow, for (A) PTH1R and Sost, (B) FoxO1, and ATF4 expression. (C) RT-qPCR analysis of RNA isolated from femoral cortical bone for OPG. The representative data of three independent experiments is presented as mean ± SD.
